# Electric and Electrochemical Microfluidic Devices for Cell Analysis

**DOI:** 10.3389/fchem.2019.00396

**Published:** 2019-06-04

**Authors:** Kaoru Hiramoto, Kosuke Ino, Yuji Nashimoto, Kentaro Ito, Hitoshi Shiku

**Affiliations:** ^1^Graduate School of Environmental Studies, Tohoku University, Sendai, Japan; ^2^Graduate School of Engineering, Tohoku University, Sendai, Japan; ^3^Frontier Research Institute for Interdisciplinary Sciences, Tohoku University, Sendai, Japan

**Keywords:** electric devices, electrochemical devices, microfluidic devices, cell manipulation, cell analysis, organs-on-a-chip

## Abstract

Microfluidic devices are widely used for cell analysis, including applications for single-cell analysis, healthcare, environmental monitoring, and organs-on-a-chip that mimic organs in microfluidics. Moreover, to enable high-throughput cell analysis, real-time monitoring, and non-invasive cell assays, electric and electrochemical systems have been incorporated into microfluidic devices. In this mini-review, we summarize recent advances in these systems, with applications from single cells to three-dimensional cultured cells and organs-on-a-chip. First, we summarize microfluidic devices combined with dielectrophoresis, electrophoresis, and electrowetting-on-a-dielectric for cell manipulation. Next, we review electric and electrochemical assays of cells to determine chemical section activity, and oxygen and glucose consumption activity, among other applications. In addition, we discuss recent devices designed for the electric and electrochemical collection of cell components from cells. Finally, we highlight the future directions of research in this field and their application prospects.

## Introduction

**Figure 1:**
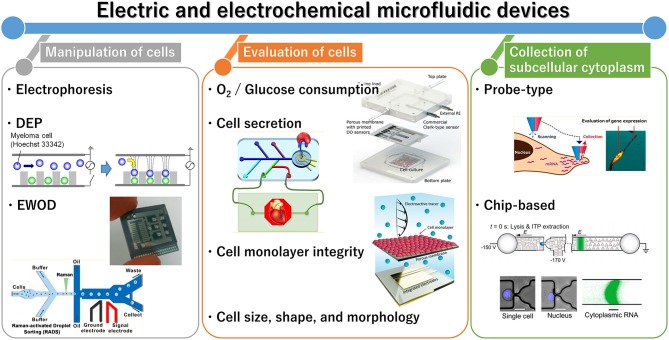
General outline of electric and electrochemical microfluidic devices for cell analysis. Reprinted with permission from Yoshimura et al. (2014); Nashimoto et al. (2016); Shin et al. (2016); Wang et al. (2017). Copyright (2014, 2016, and 2017) American Chemical Society. Reprinted with permission from Rival et al. (2014); Moya et al. (2018); Wong and Simmons (2019). Reproduced from Abdelmoez et al. (2018).

Cell analysis is essential for healthcare and environmental monitoring, and has recently benefited from the organs-on-a-chip technique that can effectively mimic organs and their microfluidics (Bhatia and Ingber, 2014; Rogal et al., 2017). Recently, a wide diversity of microfluidic devices have been adapted for cell analysis, which allows for many samples to be assessed simultaneously and for the construction of functional tissue models. Microfluidic techniques can compartmentalize, monitor, sort, collect, lyse, and culture individual cells and cell aggregates; in addition, cell-derived signals can be amplified. In cell culture, microfluidic platforms can enable cell stimulation and mimic cellular environments. Therefore, the techniques are considered superior to conventional methods such as microscopic imaging, flow cytometry, and cell culture. Although optical approaches are commonly used for evaluating cells, novel electric and electrochemical approaches are also increasingly reported. This mini-review summarizes recent advances in electric and electrochemical microfluidic devices developed for cell analysis with a range of applications, from single cells to three-dimensional (3D) cultured cells and organs-on-a-chip (Figure 1). In the first section, we summarize the microfluidic devices currently available for cell manipulation. In the second section, we discuss the common electric and electrochemical-based cell assays, followed by a description of approaches for collecting cell components. Finally, we provide a summary and discussion of future directions of research and prospects in this field.

## Manipulation of Cells and Droplets Containing Cells

In some cases, the cells are introduced from an inlet to be trapped and assayed sequentially in a microfluidic chip. In addition, droplets containing cells can be manipulated for cell lysis and polymerase chain reaction (PCR)-based assays. Electric and electrochemical approaches are widely used for all of these applications, including electrophoresis, dielectrophoresis (DEP), and electrowetting-on-dielectric (EWOD).

### Electrophoresis

Driven by the negative charges of cell membranes, cells can be moved from a microfluidic channel to an electrode applied at a positive potential. For example, yeast cells were trapped using electrophoresis, and a reporter protein was electrochemically evaluated using the electrode (Yasukawa et al., 2008; Ino et al., 2009). After the evaluation, the cells are pushed to the microfluidic channel. This allowed for sequential assays of the cells using only a single electrochemical sensor.

In particular, Capillary electrophoresis (CE) devices are widely used for cell manipulation (Mellors et al., 2010). In this case, the cells migrate via electro-osmotic flow toward the lysis intersection where the electric field is increased. Ultimately, these cells can be evaluated with several methods, including mass spectrometry (Mellors et al., 2010).

Thus, the manipulation of molecules and particles is based on their charges. The following section describes DEP, which is used for the manipulation of non-charged particles.

### DEP

DEP is a specific form of electrophoresis that is used for manipulating biosamples that contain non-charged particles (Pohl, 1951). In general, DEP involves the application of AC voltage, and the cells are pushed toward high or low intensity of electric fields by inducing positive and negative DEP, respectively, depending on the AC frequency. DEP manipulation can be used for assays of single cells (Thomas et al., 2009) to 3D cultured cells (Kanno et al., 2015c). Recently, the DEP technique was combined with a bipolar electrode system, and the cells were manipulated in a microfluidic device via electrode arrays in the absence of ohmic contacts (Anand et al., 2015). In addition, a DEP device has been used for cell paring by trapping single cells sequentially (Sen et al., 2013b; Yoshimura et al., 2014; Wu et al., 2017), and a microfluidic DEP device was applied for separation of mesenchymal stem cells (MSCs) from a cell mixture according to their dielectric properties (Yoshioka et al., 2018) and controlled cell differentiation (Yoshioka et al., 2016). Moreover, enrichment and detection of target cancer cells were performed by combining DEP manipulation and impedance measurements (Ngoc-Viet and Jen, 2018).

DEP devices have also been proposed for high-throughput droplet sorting, and single cells compartmentalized in droplets are monitored using a fluorescence method (Baret et al., 2009) and Raman spectroscopy (Wang et al., 2017).

DEP is widely used for manipulation of cells and droplets in a solution. For manipulating droplets in air, other techniques, such as EWOD, are utilized.

### EWOD

The EWOD technique can modulate the surface tension between a liquid droplet and solid substrate through application of voltage to an electrode. In this technique, individual droplets are moved along an array of electrodes. As an application of the technique for cell analysis, droplets containing cells and chemicals were manipulated in a microfluidic device for the isolation of a single cell for subsequent RNA purification and gene expression analysis (Rival et al., 2014). More recently, Sinha et al. developed a droplet-based digital microfluidic platform for gene editing (Sinha et al., 2018). The system enabled all of the necessary steps for gene editing, including cell culture, delivery, and analysis. Specifically, lung cells were cultured on the device for up to 6 days, and gene transfection and knockout procedures were performed.

In this section, we focuses on microfluidic electric and electrochemical devises for manipulating cells and droplets. These devices evaluate cells by collecting cellular components, stimulating cells, and so on. These methods are summarized in the following section.

## Evaluation of Cells

Cells in microfluidic devices are mainly measured or observed using optical techniques. For example, cells are immuno-stained in the devices, or the cells and resulting culture medium are collected from the devices, and their components are evaluated with immunostaining, enzyme-linked immunosorbent assay (ELISA), and PCR. However, these traditional techniques are time-consuming, require manual sample collection from the microfluidic system, and necessitate large working volumes. Therefore, there is strong demand for the integration of online sensor capabilities.

Recently, electric and electrochemical systems have been used for evaluating cells in microfluidic devices, and some of these key applications are highlighted below.

### Oxygen and Glucose Consumption

Since oxygen is essential for the energy metabolism of cells, it is important to determine the respiratory activity of cells. In general, platinum (Pt) electrodes are used for measuring the amount of dissolved oxygen near the cells, because O_2_ is reduced to H_2_O to obtain oxygen reduction currents at the electrodes. Some electrochemical sensors have been developed with gas-permeable membranes, known as Clark-type sensors. Moya et al. reported a liver chip integrated with multiple sensors and a porous, delicate membrane (Moya et al., 2018). The sensor electrodes were inkjet-printed along the microfluidic channel allowing for local online monitoring of oxygen concentrations. In addition to liver cells, single bovine embryos were evaluated using a microfluidic device (Wu et al., 2007). In this study, single embryos were manipulated to the sensing area with a pressure driving force, and the same force was used to collect the embryos from the microfluidic device after measurements.

Along with oxygen, glucose is a major cell energy source; thus, glucose consumption is a key parameter of cell activity. In general, enzymes such as glucose oxidase and glucose dehydrogenase can be modified with electrodes, and the resulting products of the reaction with glucose, such as H_2_O_2_ or redox mediators, are measured. Misun et al. reported a droplet array device with enzyme-modified electrodes for body-on-a-chip applications (Misun et al., 2016). This microfluidic platform is based on hanging-drop networks for the cultivation and analysis of 3D cultured cells.

In this section, we summarize the detection of consumption of target analytes. In the next section, we discuss the detection of cell secretion products.

### Cell Secretion

Many types of cell secretion products are monitored using electrochemical techniques. For example, a microfluidic chip-based perfusion system was designed to maintain viable and functioning heart tissue samples and to measure reactive oxygen species (ROS) using electrochemical methods (Cheah et al., 2010). Since ROS play a major role in cell interactions, and are related to the development and progression of many diseases, measurements of ROS levels have important clinical applications. H_2_O_2_ is a relatively stable metabolite among ROS. Therefore, it is widely utilized as a key marker of immune and inflammation processes in living cells and tissues. Inoue et al. developed an electrochemical-sensing device for the continuous monitoring of H_2_O_2_ secreted from human promyelocytic leukemia cells (Inoue et al., 2010). The electrode chip was coated with an osmium polyvinylpyridine gel polymer conjugated to horseradish peroxidase (Os-HRP), which allowed for the selective detection of extracellular H_2_O_2_ of the leukocytes. In a more recent study, Prasad et al. integrated a bare Pt electrode and an Os-HRP-modified silver electrode to monitor both the oxygen consumption and H_2_O_2_ generation of cells in a single chip, which enabled evaluation of the respiratory burst of immune cells (Prasad et al., 2016).

Lactate ions are the end product of the anaerobic pathway. Therefore, it is important to measure their accumulation in the culture medium to monitor cell metabolic activity. Bavli et al. reported a liver-on-chip device that could maintain human tissue properties for over 1 month *in vitro* under physiological conditions, and lactate was measured with enzyme-modified electrodes (Bavli et al., 2016).

Shin et al. reported a microfluidic aptamer-based electrochemical sensor for monitoring damage to cardiac organoids (Shin et al., 2016, 2017). They integrated a microfluidic bioreactor and an electrochemical biosensor in a single platform, which enabled the *in situ* detection of creatine kinase (CK)-MB as a biomarker secreted from a damaged cardiac spheroid. Electrochemical impedance spectroscopy (EIS) was adopted to the sensor system comprising a microelectrode functionalized with CK-MB-specific aptamers.

Exosomes are small (50–150 nm in diameter) vesicles secreted from various cells, and are recognized as important mediators of intracellular communication or transporters of pathogenic proteins. Moreover, exosomes have recently attracted attention as candidate biomarkers of various diseases such as cancers and metabolic disorders. Exosomes have been monitored using aptamer-based electrochemical sensors (Zhou et al., 2016). Since redox mediator-labeled probes are removed from the capture DNAs when capturing exosomes, the redox currents are decreased. In this study, exosomes were introduced from the inlets of the devices. In the future, exosomes from cells on chips will also be evaluated.

Microcapillary electrophoresis (microCE) is another approach used to analyze exosomes and extracellular vesicles. Akagi et al. developed a microCE chip and applied it to an on-chip immunoelectrophoresis assay for extracellular vesicles (EVs) of human breast cancer cells (Akagi et al., 2015). Since EVs from living bodies are heterogeneous in size, individual EVs could not be characterized by conventional methods. The microCE chip characterizes EVs according to variations in their zeta potential, which is expected to become a robust system for the sensitive profiling of EVs.

Thus, for detection of some of targets, it is important to modify electrodes. Enzymes, such as glucose oxidase, HRP, and lactate dehydrogenase are widely used to transfer electrons from target analyte to redox mediators or electrodes. In addition, several types of aptamers and antigens are modified at electrodes to capture target analytes, and the capture is electrochemically detected. These modifications are summarized in Table 1.

**Table 1:** Overview of electric and electrochemical microfluidic devices for cell analysis.

**Cell type**	**Cell culture type**	**Electric and electrochemical principle**	**Modification**	**Target**	**References**
Yeast	Single cells	Electrophoresis, amperometry	–	Reporter gene	Yasukawa et al., 2008; Ino et al., 2009
Blood cell	Single cells	CE	–	Cellular component	Mellors et al., 2010
HeLa	Single cells	DEP	–	Cell trapping	Thomas et al., 2009
ES cell	Spheroids	DEP, amperometry	–	Alkaline phosphatase	Kanno et al., 2015c
B-cells	Single cells	DEP with bipolar electrodes	–	Cell attraction and repulsion	Anand et al., 2015
3T3 cell, ES cell, MSC, myeloma, HeLa	Single cells	DEP	–	Cell pairing	Sen et al., 2013b; Yoshimura et al., 2014; Wu et al., 2017
MSC	Single cells	DEP	–	Isolation	Yoshioka et al., 2018
MSC	Single cells	DEP	–	Cell differentiation	Yoshioka et al., 2016
Lung cancer cell	Single cells	DEP, impedance	–	–	Ngoc-Viet and Jen, 2018
HaCaT	Single cells	EWOD, qPCR	–	Gene analysis	Rival et al., 2014
Hepatocyte	Monolayer	Amperometry	–	Dissolved oxygen	Moya et al., 2018
Bovine embryo	Single embryo	Amperometry	–	Dissolved oxygen	Wu et al., 2007
Human colon carcinoma cell	Spheroid	Amperometry	Enzyme	Lactate, glucose	Misun et al., 2016
Heart tissue	Spheroid	Cyclic voltammetry	–	ROS	Cheah et al., 2010
Leucocyte	Cells	Amperometry	Redox polymer, enzyme	H_2_O_2_	Inoue et al., 2010
Human monocytic leukemia	Cells	Amperometry	Redox polymer, enzyme	Oxygen, H_2_O_2_	Prasad et al., 2016
HepG2/C3A cell	Spheroid	Amperometry	Enzyme	Glucose, lactate	Bavli et al., 2016
ES cell-derived cardiomyocyte, primary hepatocyte	Spheroid	Impedance	Antigen	Creatine kinase MB, cell secretome	Shin et al., 2016, 2017
Breast cancer cell	–	Electrophoresis	–	Extracellular vesicles	Akagi et al., 2015
MDCK-2, bEnd.3, C2C12	Monolayer	TEER	–	Cell monolayer integrity	Douville et al., 2010
b.End3, astrocyte, pericyte	Multi-layer	TEER	–	Blood–brain barrier	Wang et al., 2016
Primary human airway epithelial cell	Monolayer	TEER	–	Epithelial barrier function	Henry et al., 2017
Endothelial cell, cardiomyocyte	Monolayer	TEER	–	Vascular permeability and cardiac function	Maoz et al., 2017
Primary porcine aortic endothelial cell	Monolayer	Square wave voltammetry	–	Cell monolayer permeability	Wong and Simmons, 2019
Macrophage, mast cell	Cells	Impedance	–	Cell-cell interaction	Jiang et al., 2016
Human cardiac spheroid	Spheroid	EIS	–	–	Schmid et al., 2016
Bacterial cell	Single cells	Ionic current	–	Size	Yasaki et al., 2018
Breast epithelial cell	Monolayer, spheroid	Electric cell lysis	–	mRNA	Nashimoto et al., 2007
Embryonic stem cell	Spheroid	Electric cell lysis	–	mRNA	Ito et al., 2016
Lymphoma, Human myeloid leukemia	Single cells	Electric cell lysis	–	mRNA	Subramanian Parimalam et al., 2018
ES cell, HeLa, 3T3	Single cells	Electrochemical syringe	–	mRNA	Nashimoto et al., 2016

*ES cell, Embryonic stem cell; 3T3 cell, fibroblast cell line; HeLa, cervical cancer cell; HaCaT, human keratinocyte; HepG2/C3A cell, Human hepatocellular carcinoma; MDCK, Madin-Darby canine kidney cell; b.End3, brain-derived endothelial cell; C2C12, mouse myoblast cell*.

In the first half of this section, we summarize the detection of consumption and secretion of analytes. In contrast, it is important to measure cell shape and morphology for cell evaluation. In the second half, we discuss their detection.

### Cell Monolayer Integrity

Trans-epithelial electrical resistance (TEER) is a widely used parameter for evaluating *in vitro* barrier tissue integrity (Elbrecht et al., 2016). TEER measurements are performed by applying an AC voltage at electrodes set on both sides of a cell monolayer, and the voltage and current are measured to calculate the electrical resistance of the barrier. Takayama's group evaluated epithelial and endothelial barriers in a microfluidic chip using TEER measurements (Douville et al., 2010). In addition, a blood–brain barrier (BBB) model was evaluated with this approach (Wang et al., 2016). Ingber's group also described a microfluidic device containing electrodes for assessing lung chips (Henry et al., 2017). In addition to enabling the real-time, non-invasive monitoring of barrier functions, multi-electrode arrays (MEAs) were combined with TEER measurements for heart-on-a-chip (Maoz et al., 2017).

Similar to TEER measurements, an electrochemical permeability assay was reported for evaluating cell monolayer permeability (Wong and Simmons, 2019). In this case, the ubiquitous fluorescent tracer was replaced with an electroactive tracer, and the barrier function of endothelial cells was assessed by monitoring the diffusion of the electroactive tracer across a cell monolayer.

### Cell Size, Shape, and Morphology

Impedance detection has also been applied for evaluating the allergic response in a microfluidic device. RBL-2H3 mast cells and ANA-1 macrophages were co-cultured and their allergic response to a stimulus was observed (Jiang et al., 2016). Moreover, Schmid et al. combined EIS with a microfluidic hanging-drop platform for monitoring spheroid sizes and contractions of human cardiac spheroids (Schmid et al., 2016).

Ion currents via nano- or micropores are measured for the electrical discrimination of various biomolecules, cells, bacteria, and viruses. Yasaki et al. reported a rational methodology that can detect samples within a particle volume of 0.01% of the pore volume by measuring the transient current generated in a microfluidic bridge circuit (Yasaki et al., 2017). The device was subsequently applied for the size detection of bacterial cells (Yasaki et al., 2018).

Thus, we discuss cell evaluation techniques in this section. In contrast, it is important to obtain intracellular information. In the following section, we summarize the techniques used for collection of subcellular cytoplasm.

## Collection of Subcellular Cytoplasm

We previously reviewed the progress in intracellular electrochemical sensing techniques (Ino et al., 2018b). Here, we focus on two main types of electric and electrochemical microfluidic devices for lysing cells and collecting components of cells by applying pulse voltage.

### Probe-Type Microfluidic Devices

A probe-type microfluidic device with a Pt-ring electrode at its tip was used to collect the cytosol of a single adherent cell on an ITO electrode, and cellular mRNA was analyzed at the single-cell level (Nashimoto et al., 2007). The bias was applied between the Pt and ITO electrodes. To collect the mRNA of single cells in 3D cultured cells, a double-barrel carbon probe was developed (Ito et al., 2016). The device was used to measure local gene expression levels during sprouting angiogenesis in embryonic stem cell-derived embryoid bodies.

Laforge et al. reported an electrochemical syringe containing organic and liquid solutions for manipulating aL–fL-scale volumes (Laforge et al., 2007). The interface between the organic and liquid solution in a pipet was moved by applying a potential. Previously, we combined a syringe with scanning ion conductance microscopy (SICM) for measuring cellular morphology and collecting the cytosol from living cells (Nashimoto et al., 2016).

### Chip-Based Devices

Shintaku's group reported a microfluidic device containing a hydrodynamic trap for electrical lysis (Subramanian Parimalam et al., 2018). All on-chip processes were completed in <5 min. In addition, a microfluidic system was designed to physically separate nuclear and cytoplasmic RNAs from a single cell, allowing for separate analyses of these RNAs (Abdelmoez et al., 2018).

Since electrical techniques can provide precise control of applied potentials, they enable the collection of subcellular cytoplasm at a single cell level. By combining these techniques with microfluidic devices, the throughput of the collection will be improved.

## Conclusion and Prospects

In this mini-review, we have presented an overview of recent research progress in electric and electrochemical microfluidic devices for cell analysis. These applications and devices are summarized in Table 1.

In typical microfluidic devices, only a few electrodes or electrochemical sensors are incorporated. However, the future goal of microfluidic device design is to provide multiplex assays and electrochemical imaging of cell activity. Electrochemical imaging enables chemical mapping using electrode arrays. For these purposes, it is important to incorporate several individual and addressable electrodes. Although numerous reports have described such devices in which the electrodes are simply arranged, with useful applications in cell analysis, including neurons (Kasai et al., 2005), the areas of the leading electrodes and connector pads are large, which limits the density and number of electrodes that can be incorporated. Recently, several kinds of integrated electrode arrays have been developed to overcome this limitation (Ino et al., 2012, 2014; Kätelhön et al., 2014; Kanno et al., 2015c; Zhang et al., 2017). These systems can provide electrochemical images consisting of 2D current values. Compared to assays using a single electrode, spatial information can be obtained and/or multiplex sensing can be performed with an integrated system.

Moreover, in the future, electrode array devices will be combined with microfluidic systems. In particular, large-scale integration (LSI) technology will be used for incorporating many addressable electrodes into microfluidic devices. Indeed, LSI devices have already been used for electrochemical imaging (Inoue et al., 2012, 2015; Sen et al., 2013a, 2014; Abe et al., 2015, 2016; Kanno et al., 2015a,b, 2016, 2017; Ino et al., 2017, 2018b,c,d, 2019a,b). Since signal amplifiers and switching elements are incorporated into the sensing area, highly sensitive sensors can be incorporated with high density. In addition, measurement modes such as amperometry and potentiometry can be selected separately for each sensor, allowing for multiplex analytes to be simultaneously measured. Further, electrochemical imaging in microfluidic devices will be combined with optical imaging analysis using mathematical approaches (Kato et al., 2016; Nagasaka et al., 2017) to improve their analytical features.

Since electrodes are inevitably fouled during the measurement, long-term assays remain a challenge of this technology. For example, measurement of oxygen consumption using non-modified electrodes can only be continuously performed for 2 days. For long-term monitoring, the electrodes should be modified with certain materials, or the electrodes will need to be changed every 2 days. For this purpose, the attachment and connectors should be improved. In addition, enzymes might be unsuitable for long-term measurement because their activity gradually decreases. To solve these problems, non-enzyme electrochemical sensors using metal nanoparticles and porous surfaces have been reported (Niu et al., 2013). The sensors are expected to be widely used for cell analysis in microfluidic devices.

For multiplex assays, individual sensors should be modified with different materials such as glucose oxidase and HRP. For this purpose, electrodeposition is a useful technique for addressable and individual electrode arrays. Since electrodeposition is also applied for hydrogel formation (Ozawa et al., 2013a,b, 2016, 2017; Ino et al., 2018a,d; Taira et al., 2018, 2019; Li et al., 2019), biomaterials including cells are easily patterned only on the target electrodes.

In general device fabrication, photolithography, and metal-sputtering techniques are typically used. In contrast, it is expected that printing techniques will become more widely used in future applications, including inkjet printing (Bachmann et al., 2017) and printed organic transistor-based enzyme sensors (Mano et al., 2018). Lind et al. reported cardiac physiological devices designed via multilateral 3D printing (Lind et al., 2017). Curto et al. reported an organic transistor platform with integrated microfluidics for in-line multi-parametric *in vitro* cell monitoring (Curto et al., 2017). Moreover, printed carbon electrodes have been reported (Shitanda et al., 2018) for reducing the sensor cost. Although the majority of probe and chip devices are currently made from glass, silicon, and plastic materials, paper devices (Yang et al., 2017; Akyazi et al., 2018; Jin et al., 2018) are also widely used for low-cost assays. In the future, paper-based devices are expected to be more commonly used for on-chip cell analysis.

Nanodevices are also increasingly used for cell analysis in microfluidic chips. For example, nanopores were used for measuring oligonucleotides and microvesicles via detection of ion currents when passing through the pores (Anderson et al., 2015; Kawano, 2018). For highly sensitive and selective assays, a redox cycling system was used in nanofluidic and nanocavity devices including two sets of electrodes in close proximity to one another (Lemay et al., 2013; Kätelhön et al., 2014; Kanno et al., 2015c). A redox species generated at one electrode diffuses to the other electrode, which generates an electrochemical reaction to regenerates the original species, resulting in signal amplification. In addition to these nanosystems, a pyrolyzed carbon mesh was integrated into a microchannel for a good redox cycling efficiency (Lee et al., 2018). These systems are expected to be incorporated into microfluidic devices for improved cell analysis.

In this review, we described the electric and electrochemical devices for heart-on-a-chip, liver-on-a-chip, carcinoma microtissue models, and so on. Since lung-on-chip (Huh et al., 2010) and gut-on-a-chip (Kim et al., 2012) devices have attracted considerable attention, electric and electrochemical systems will be incorporated into these organ-on-a-chip devices.

In this review, we discuss the cell manipulation for cell analysis in microfluidic devices. In future, these techniques will also be used for fabrication of functional tissue models. Some studies have reported the fabrication of tissue models for manipulating cells and scaffold materials using DEP (Ramón-Azcón et al., 2012, 2013); these approaches will be combined with microfluidic techniques.

We believe, the presented electric and electrochemical techniques have high expectations to broaden the possibilities of microfluidic devices and cell analysis.

## Author Contributions

All authors wrote the manuscript. KoI and HS supervised preparation of the paper.

### Conflict of Interest Statement

The authors declare that the research was conducted in the absence of any commercial or financial relationships that could be construed as a potential conflict of interest.
